# Development of Lateral Flow Immunochromatographic Assays Using Colloidal Au Sphere and Nanorods as Signal Marker for the Determination of Zearalenone in Cereals

**DOI:** 10.3390/foods9030281

**Published:** 2020-03-04

**Authors:** Mingfei Pan, Tianyu Ma, Jingying Yang, Shijie Li, Shengmiao Liu, Shuo Wang

**Affiliations:** 1 State Key Laboratory of Food Nutrition and Safety, Tianjin University of Science & Technology, Tianjin 300457, China; panmf2012@tust.edu.cn (M.P.); maty1128@126.com (T.M.); yangjy0823@126.com (J.Y.); lsj930427@126.com (S.L.); lsm20000711@outlook.com (S.L.); 2 Key Laboratory of Food Nutrition and Safety, Ministry of Education of China, Tianjin University of Science and Technology, Tianjin 300457, China

**Keywords:** zearalenone, immunochromatographic assay, colloidal Au sphere, Au nanorods, cereals

## Abstract

This paper describes the development of lateral flow immunochromatographic assays (ICAs) using colloidal Au sphere (SP) and nanorods (NRs) as signal markers for the determination of zearalenone (ZEN) in cereals. The developed ICAs can detect the analyte ZEN within a short time (10 min), and achieve lower limit of detection (LOD). This is the first time that the AuNRs are used as signal probe in immune test strip for ZEN detection. For colloidal AuSP immunochromatographic analysis (AuSP-ICA), the LODs in solution and spiked cereal sample were 5.0 μg L^−1^ and 60 μg kg^−1^, and for AuNRs immunochromatographic analysis (AuNRs-ICA) the two LODs achieved 3.0 μg L^−1^ and 40 μg kg^−1^, respectively. These two proposed ICAs have minor cross-reaction to the structural analogs of ZEN, and no cross-reactivity with aflatoxin B_1_, T-2 toxin, ochratoxin A, deoxynivalenol, fumonisin B_1_. Both of the developed ICAs can specifically and sensitively detect ZEN in cereals, providing an effective strategy for rapid screening and detection of ZEN in a large number of food samples.

## 1. Introduction

Zearalenone (ZEN), also known as F-2 toxin, is a secondary metabolite produced by Fusarium, which mainly contaminates corn, wheat, and other cereals [1,2]. ZEN has a similar structure with the endogenous estrogen and can competitively bind to the estrogen receptor, which makes the hormone of animal body maladjusted and finally destroys the reproductive system [3,4]. The contamination range of ZEN in grain is very wide. It cannot only enter the human body through direct or indirect intake, but can be transmitted to the fetus through placenta. ZEN with high exposure and high accumulation concentration can inhibit the activity of enzyme and stimulate the growth of breast cancer cells. Modern research has confirmed that ZEN has typical immunotoxicity, genotoxicity, and reproductive toxicity, which seriously threatens people’s health [5,6,7]. As a result, many countries and organizations have stipulated the maximum residue limits (MRLs) for ZEN in cereals (the United States: 20–100 µg kg^−1^ for cereals [8]; European Union: 350 µg kg^−1^ in raw maize products [9]; China: 60 µg kg^−1^ in cereals and cereals products [10]). Therefore, the development of effective methods for ZEN detection in various foods is of great significance for protecting the health of people.

At present, the analysis strategy for ZEN in foods is mainly the accurate and precise analysis using analytical instruments based on chromatographic separation and mass spectrometry, including high-performance liquid chromatography, gas chromatography, and related analytical methods combined with mass spectrometry [11,12,13]. This type of methods is widely recognized by testing agencies because of its high accuracy, good sensitivity, and very high reproducibility. On the other hand, because of the limit of large analytical instruments, these methods require a relatively cumbersome pre-treatment process to deal with complex food matrices, resulting in having a certain distance in terms of detection throughput and convenience. As known, the immunoassays based on the specific binding of antigen-Ab have obvious advantages in the rapid screening and analysis of a large number of samples. Established relatively mature immunoassays to ZEN included enzyme-linked immunosorbent assay (ELISA) [14,15] and colloidal gold [16,17,18] and fluorescent [19,20] immunochromatographic test strips. The ELISA with high sensitivity and specificity does not require the high purity of tested samples, but needs skillful operators. The lateral flow immunochromatographic assays strips (ICAs) are easy to operate, convenient to carry, and can directly visualize qualitative or semi-quantitative analysis of the target, having remarkable application prospects for the detection of toxic and harmful substances in foods [21,22]. Based on different markers such as colloidal Au spheres (SP) [23,24,25], fluorescent microspheres [26,27], quantum dot [28,29], etc., ICAs products (test strips) are easy to use and inexpensive, making them have very important applications in fast, on-site screening of large numbers of food samples. Au nanorods (AuNRs) is one of the more precious metal nanomaterials studied in recent years. The different aspect ratios of AuNRs enable them to show different absorption spectra or different colors, which is very suitable for rapid screening of visualization. There are some reports about the application of AuNRs in toxin and mycotoxins in food products [30,31,32]. The AuNRs was applied as a signal output in ELISA or biosensor, achieving qualitative and quantitative detection for the targets. So far, the research on AuNRs has mostly focused on analysis strategies based on fluorescent signals [33] and biological imaging [34], but less research on the visible analysis [35].

In this study, two Au nanomaterials-colloidal Au SP and NRs were successfully prepared and used to develop two immunochromatographic test strips for the visual detection of ZEN in cereals. Based on the physical and chemical properties of colloidal Au SP and NRs, colloidal AuSP immunochromatographic analysis (AuSP-ICA) and AuNRs immunochromatographic analysis (AuNRs-ICA) provide an effective analysis strategy for monitoring and analysis of this toxin in food products. Additionally, this is the first time to detect ZEN in food products using AuNRs as the signal marker.

## 2. Materials and Methods

### 2.1. Materials

The anti-ZEN monoclonal antibody (anti-ZEN-Ab, 1 mg mL^−1^) and coating antigen, the conjugate of ZEN-ovalbumin (ZEN-OVA, 2.94 mg mL^−1^), were purchased from Shandong Lvdu Biotechnology Co. Ltd. (Shandong, China). The chloroauric acid (HAuCl_4_) and trisodium citrate for the synthesis of Au SP and NRs were obtained from Sigma-Aldrich (St. Louis, MO, USA). The goat anti-mouse Ab (4 mg mL^−1^), aflatoxin B_1_ (AFB_1_), ZEN, and the analogues (α-zearalenol, α-zearalanol, β-zearalenol, zearalanone, β-zearalanol) (1.0 mg L^−1^) were also purchased from Sigma-Aldrich (St. Louis, MO, USA). Other mycotoxins T-2, ochratoxin A (OTA), deoxynivalenol (DON), and fumonisin B_1_ (FB_1_) were purchased from Toronto Research Chemicals (Toronto, Canada). Tris, sucrose, polyvinylpyrrolidone (PVP), poloxamer 18 solution (F68), TritonX-100, SDS-L, and polyethylene glycol (PEG_200_) for the preparation of gold-label working buffer were purchased from Sangon Biotech Co., Ltd. (Shanghai, China). Hexadecyl trimethyl ammonium bromide (CTAB, 99%) was purchased from Solarbio (Beijing, China). Silver nitrate (AgNO_3_) and ascorbic acid for NRs synthesis were obtained from the Sinopharm Chemical Reagent Co., Ltd. (Shanghai, China).

Nitrocellulose (NC) membranes (HF90, HF135, and HF180 with capillary flow rates of 90 s/4 cm, 135 s/4 cm and 180 s/4 cm, respectively) were purchased from Millipore Corporation (Bedford, MA, USA). Sample pad, conjugate pad and absorbent pad, and polyvinyl chloride (PVC) sheets were purchased from Kinbio Tech Co. (Shanghai, China). Single-channel pipettes (2.5–1000 μL) were obtained from Thermo Corporation (USA). A vortex machine (HQ-60) was purchased from North Tongzheng Biotechnology Development Company (Beijing, China). Milli-Q Ultrapure Water System was purchased from Milli-Q Millipore, (Bedford, MA, USA). The UV-visible spectrophotometer (Cary 50 Bio) was obtained from Varian (California, USA). Three-dimensional film-gold spraying instrument (HM3030) and the microcomputer automatic cutting machine (ZQ2002) were purchased from Shanghai Jinbiao Biotechnology Co., Ltd. (Shanghai, China) for the preparation of test strips.

### 2.2. Preparation of Colloidal Au SP and NRs

Colloidal AuSP was synthesized according to the previously reported synthesis method with some new modifications [25]. Total of 99.0 mL of water and 1.0 mL of HAuCl_4_ (1%) were added into a glassy bottle, and the mixture was stirred and heated at 100 °C. When the mixture begins to boil, 2.5 mL of trisodium citrate (1%) was added immediately. When the mixture boils violently, the heating is stopped. When the color of the mixture turns to claret-red, the mixture was boiled for 15 min, cooled to the room temperature, and stored at 4 °C for later use.

AuNRs was synthesized using seed-mediated method, and some improvements were made [36,37]. Total of 1.0 mL of CTAB (0.2 mol L^−1^) and 1.0 mL of HAuCl_4_ (0.5 mmol L^−1^) were mixed in a glassy bottle, and 0.12 mL of NaBH_4_ (0.01 mol L^−1^) was further added into the above mixture, and stirred gently for 2 min (the color of the mixture changes from yellow to brownish yellow) as the seed liquid. After 40.0 mL of CTAB (0.2 mol L^−1^) and 40.0 mL of HAuCl_4_ (1 mmol L^−1^) were evenly mixed, 0.8 mL of AgNO_3_ (0.01 mol L^−1^), 0.64 mL of ascorbic acid (0.1 mol L^−1^), and 0.8 mL of HCl (2 mol L^−1^) were added in order, and mixed well as the growth liquid. Take 0.28 mL of seed liquid into the above growth liquid, and finally put the mixture into a 30 °C water bath for 12 h without stirring. The mixture was centrifuged at 10,000 rpm for 20 min to remove CTAB. The AuNRs product was reconstituted with 40.0 mL of ultrapure water for use.

### 2.3. Preparation of Immune Signal Probe

**Figure 1:**
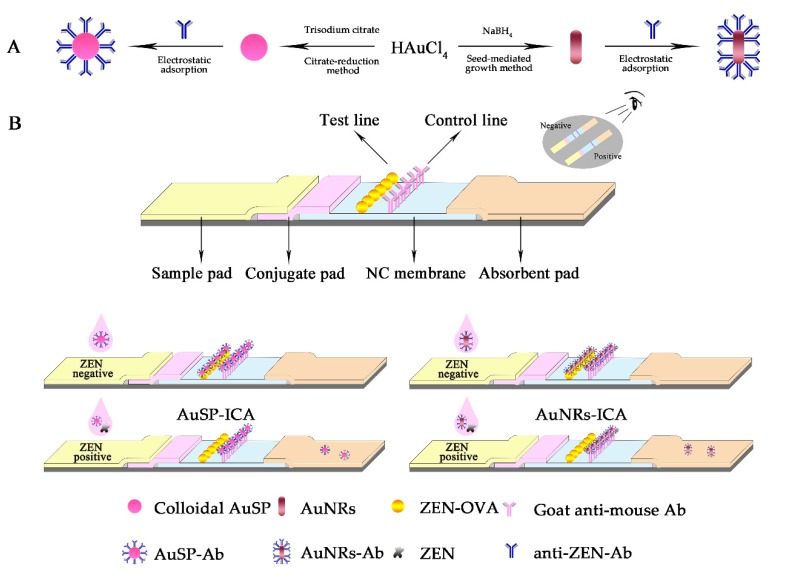
Schematic of AuSP-ICA and AuNRs-ICA. (**A**) The synthesis of AuSP and AuNRs and the coupling process with anti-ZEN-Ab; (**B**) structure of the immunochromatographic assay (ICA) strip and the test procedure for the ICAs.

The obtained colloidal Au SP and NRs were employed to prepare the immune signal probes (AuSP-Ab and AuNRs-Ab). The anti-ZEN-Ab was modified onto the AuSP and AuNRs by electrostatic adsorption and the whole process is as follows (Figure 1A). First, a certain volume (0, 1.0, 2.0, 3.0, 4.0, and 5.0 μL) of K_2_CO_3_ (0.2 mol L^−1^) was mixed with 0.8 mL of the colloidal AuSP or AuNRs to adjust the pH, and then the anti-ZEN-Ab (1.0 mg mL^−1^) at a volume of 1.0, 2.0, 3.0, 4.0, and 5.0 μL was also added and incubated at room temperature for 1 h. Then, 8.0 μL of PEG-20000 (10%) and 16.0 μL of BSA (20%) were added and incubated for 0.5 h. The obtained mixture was centrifuged at 8000 rpm for 20 min at 4 °C. The solid product was further reconstituted with 80 μL of gold-label working buffer at pH 7.0.

### 2.4. Preparation and Test Procedure of Immunochromatographic Test Strips

Immunochromatographic test strip was composed of four parts, from left to right (the sample pad, conjugate pad, NC membrane, and absorbent pad) (Figure 1B). These four parts were sequentially pasted on the PVC sheets. Goat anti-mouse Ab was fixed on the control line (C line) at 0.6 μL cm^−1^, and the conjugate of ZEN-OVA was fixed on the test line (T line) at 0.6 μL cm^−1^ for 0.5 cm to C line. Put the sliced membrane into 37 °C incubator and incubate it overnight, then cut into strips with a width of 0.38 cm. For the AuSP and AuNRs-ICA test strips, 100 μL of ZEN solution (standard or sample), 2.0 μL of signal probe (AuSP-Ab or AuNRs-Ab) solution, and 12 μL of gold-label working buffer were fully mixed in a tube and uploaded onto the sample pad. The results were observed after 10 min.

### 2.5. Sample Analysis

Total of 5.0 g of cereal sample (corn and wheat powder) was accurately weighed, passed through a 0.05–2 mm sieve, and placed into a 50 mL glass centrifuge tube. A certain concentration of ZEN standard solution was added to control ZEN concentration in samples ranging from 0 to 80 μg kg^−1^. After standing for 5 min, the mixture was shaken for 15 min by a vortex machine. A total of 10.0 mL of the solution containing methanol and PBS (5/5, *V*/*V*) for sample extraction was added into the tube. The mixture was centrifuged at 8000 rpm for 20 min and the supernatant was filtered using a 0.22 µm filter, and the filtrate was diluted eight times using sample diluent for visual analysis using the AuSP and AuNRs test strips.

## 3. Results and Discussions

### 3.1. Synthesis and Characterization of AuSP and AuNRs

**Figure 2:**
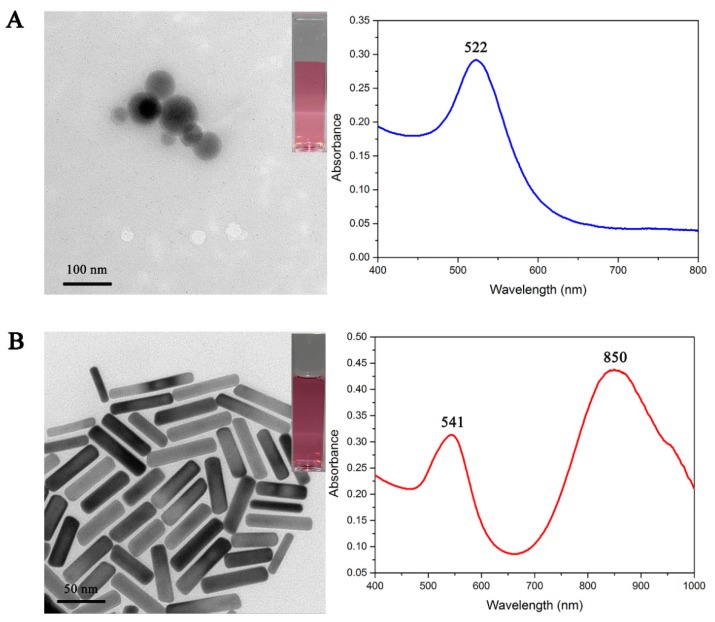
Transmission electron microscope (TEM) image and UV spectra of the prepared Au nanomaterials (**A**) colloidal AuSP (**B**) AuNRs.

Colloidal AuSP was synthesized according to a classical method of trisodium citrate reduction. Figure 2A shows the images of digital and transmission electron microscope (TEM) of the prepared colloidal AuSP. It was clearly observed that the size of colloidal AuSP was uniform with an average particle size of 20.95 nm. From the digital image, the solution of colloidal AuSP was claret-red without obvious precipitation. The absorption curve of UV spectra was smooth, and the maximum absorption peak at 522 nm was narrow and high, which indicated the uniform particle size of the prepared colloidal AuSP.

The AuNRs was prepared according to the classical seed-mediated method. The difference is that by controlling the amount of HCl (0.8 mL, 2 mol L^−1^), the irregular shape of AuNRs is reduced. Figure 2B shows that the size of the prepared AuNRs was uniform and the aspect ratio was about 3.98. The AuNRs solution is shown in claret-colored. The UV-visible spectra of AuNRs indicated that the centers of the transverse and longitudinal SPR peaks are located at 541 nm and 850 nm, respectively.

### 3.2. Optimization of the Parameters of ICAs

The experimental parameters including the pH for probe coupling, the anti-ZEN-Ab coupling ratio, the pore size of the membrane, the amount of goat anti-mouse Ab on the C line, the amount of coated antigen (ZEN-OVA) on the T line, and the buffer solution were optimized in detail to construct an effective immunochromatographic method for ZEN analysis.

The optimal experimental conditions are determined by the three following factors: (a) No gathering during the coupling process; (b) having color on the test strip during the detection process; (c) when the negative sample is detected, the color of the C and T lines are consistent. The following experimental conditions were found to obtain the best results: (1) Optimal probe coupling pH: 3.0 μL of K_2_CO_3_ (0.2 mol L^−1^) for AuSP-ICA, 4.0 μL of K_2_CO_3_ (0.2 mol L^−1^) for AuNRs-ICA; (2) the best anti-ZEN-Ab coupling ratio: 5.0 μL anti-ZEN-Ab for AuSP-ICA (the mass ratio of HAuCl_4_/Ab was 16/1), 5.0 μL anti-ZEN-Ab for AuNRs-ICA (the mass ratio of HAuCl_4_/Ab was 32/1); (3) the optimal pore size of the membrane: HF135s NC membrane for AuSP-ICA and AuNRs-ICA; (4) the amount of goat anti-mouse Ab on C line: 0.16 μg cm^−1^ for AuSP-ICA and AuNRs-ICA; (5) optimal amount of ZEN-OVA on T line: 0.16 μg cm^−1^ for AuSP-ICA and AuNRs-ICA; (6) optimum determination buffer: for AuSP-ICA and AuNRs-ICA, pH 7.4 phosphate buffer saline (PBS) buffer (0.01 mol L^−1^).

### 3.3. The Sensitivity and Specificity of the AuSP-ICA and AuNRs-ICA

**Figure 3:**
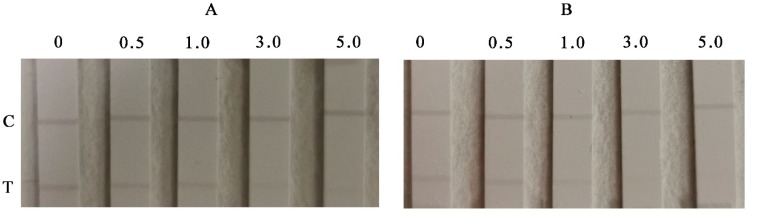
Results of zearalenone (ZEN) detection using AuSP-ICA and AuNRs-ICA strips. (**A**) colloidal AuSP (**B**) AuNRs; tested ZEN concentrations in PBS buffer (pH 7.4): 0, 0.5, 1.0, 3.0, and 5.0 μg L^−1^.

Both the developed ICAs in this work are based on the principle of competitive reaction between antigen and Ab. The detection process is described as follows: The ZEN sample testing solution and the immune signal probes (AuSP-Ab and AuNRs-Ab) were mixed evenly and uploaded onto the sample pad. The mixture is chromatographed gradually toward the absorbent pad because of the capillary forces. For negative samples, the red signal probes were intercepted by ZEN-OVA fixed on the T line which appeared red, and the excess probes continued to be chromatographed upwards and captured by the goat anti-mouse Ab fixed on the C line which was also red. For positive samples, ZEN in the sample solution and ZEN-OVA on the T line competitively bound to the signal probe, causing the T line to become lighter in color. As the ZEN content in the sample solution increased, the color of the T line gradually disappeared, and only the C line was observed to be red. The ZEN concentration that caused the red or purple disappearance of the T line were defined as the limits of detection (LOD) of these two assays. If the C line disappeared, the T line was in color, or both the C and T lines disappeared during testing, it indicated that the test strips were invalid. Under the optimized conditions above, two ICAs have been developed as different sensitivity and semi-quantitative detection (Figure 3)**.**

For AuSP-ICA, the red color of the T line gradually became lighter as the ZEN concentration increases, and eventually disappeared when the ZEN concentration was 5.0 μg L^−1^ (Figure 3A). For AuNRs-ICA, the red color of the T line was also shallower as the ZEN concentration increases gradually and eventually disappeared when the ZEN concentration was 3.0 μg L^−1^ (Figure 3B). Therefore, the LODs of the AuSP-ICA and AuNRs-ICA in visual analysis were 5.0 μg L^−1^ and 3.0 μg L^−1^, respectively. Based on the same amount of anti-ZEN-Ab and ZEN-OVA in the test, the developed AuNRs-ICA was more sensitive than the AuSP-ICA. This may be due to the unique structure of AuNRs and their different adsorption ability to antibodies, which also means that AuNRs have broad application prospects in rapid visual detection.

Ten mycotoxins including five structural analogs of ZEN (α-zearalenol, β-zearalenol, α-zearalanol, β-zearalanol, zearalanone), AFB_1_, OTA, T-2 toxin, FB_1_, and DON were selected to evaluate the specificity of the two developed ICAs in the work. The chemical structure for ten mycotoxins is shown in Table S1. From the results, when the concentration of zearalanone was 300 μg L^−1^, the T line on the AuSP-ICA and AuNRs-ICA test strip disappeared. But for four other structural analogs of ZEN, the concentration should achieve 50 μg L^−1^ to cause the T line to disappear. The concentrations of AFB_1_, OTA, T-2 toxin, FB_1_, and DON were 1000 μg L^−1^, the color of T line can still be observed. These results indicated that the proposed AuSP-ICA and AuNRs-ICA have minor cross-reactions occurring between ZEN and five other structural analogs, and no cross-reactivity with AFB_1_, OTA, T-2 toxin, FB_1_, DON.

### 3.4. Detection of ZEN in Practical Samples

The matrix components and organic reagents in the sample extract would affect the specific binding of ZEN-OVA and anti-ZEN-Ab in the detection process. A series of ZEN solutions were constructed using different sample diluent and tested by the AuSP-ICA and AuNRs-ICA to eliminate the potential matrix effects. By comparison of the test results obtained using sample extract at different diluted times, it was demonstrated that the eight times diluted sample extract can obtain the elimination line compared with that in PBS buffer. That means the matrix component and organic reagent in the extract did not interfere with the specific binding of ZEN-OVA and anti-ZEN-Ab. As a result, the eight times diluted sample extract using the PBS buffer of pH 7.4 was chosen for further research.

**Figure 4:**
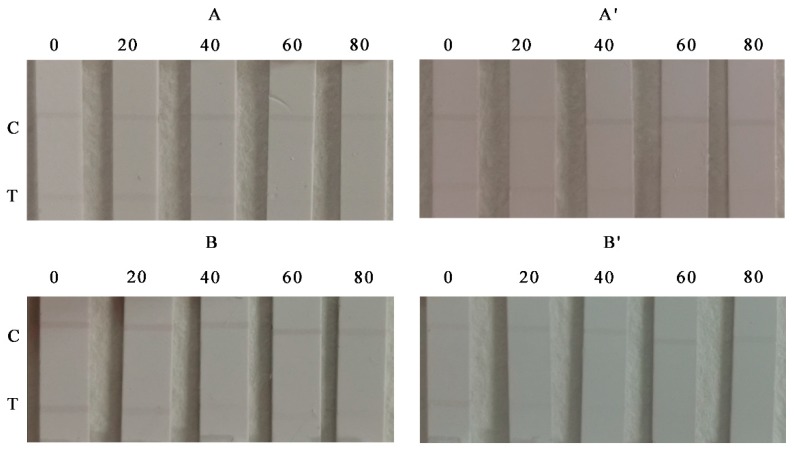
Results of ZEN detection in spiked cereal samples using AuSP-ICA (**A**: corn; **B**: wheat) and AuNRs-ICA (**A’**: corn; **B’**: wheat) strips. Spiked ZEN concentrations in cereal samples: 0, 20, 40, 60, and 80 μg kg^−1^.

Cereal samples (including corn and wheat powder) were purchased from a local supermarket. Figure 4 shows the detection results of AuSP-ICA and AuNRs-ICA strips for visual detection of ZEN in spiked food samples at various concentrations of 0, 20, 40, 60, and 80 μg kg^−1^. It is obviously shown that for AuSP-ICA, when the ZEN concentration in cereal samples was 60 μg kg^−1^ (LOD), the T line disappeared (Figure 4A). The AuNRs-ICA strip can obtain the elimination line at the ZEN sample concentration of 40 μg kg^−1^ (LOD)**,** indicating the AuNRs-ICA strip has better sensitivity and wider detection range than the AuSP-ICA (Figure 4A’). Figure 4 B and B’ shows the test results using AuSP-ICA and AuNRs-ICA to detect ZEN in wheat samples, which have the same visual LODs with corn. These results demonstrated that the developed AuSP-ICA and AuNRs-ICA strips have good response to the analyte ZEN and can be applied in the detection of ZEN in practical samples. These results also demonstrated that it is feasible to employ AuNRs material as signal probe marker for the detection of small molecule contaminants in the ICAs. Table 1 shows a comparison of the results from different reported methods, signifying the merits of the developed AuSP-ICA and AuNRs-ICA in rapid screening and detection of ZEN in large number of samples.

## 4. Conclusions

This study has developed two kinds of visual immunoassay test strips based on the colloidal AuSP and AuNRs with good specificity and high sensitivity for ZEN toxins in cereals. This is the first time that the AuNRs are used as signal probe in immune test strip for rapid detection of ZEN, which has proved the feasibility and merits of AuNRs as a signal probe for visual detection. It also provided the application reference for the research in the field of rapid food safety. Although the established two ICA test strips can only provide qualitative or semi-quantitative analysis results, the entire detection process can be completed in 10 min, and the sensitivity provided can meet the required maximum residue limit. This provides an effective technical support for the rapid screening and detection of ZEN toxin in cereals.

## Figures and Tables

**Table 1:** Results of the comparison of different reported methods for ZEN detection.

Methods	LOD (μg L^−1^ or μg kg^−1^)	Test Time	Samples	References
Ultra HPLC-MS/MS	0.05	7 min	Chinese yam, coix seed	[11]
GC-MS	0.01	1 h	Vegetable oil	[13]
Electrochemical immunosensor	1.5 × 10^−4^/0.25	More than 1 h	Maize/Beer, wine	[38]/[39]
ELISA	0.04/0.15/4 × 10^−3^	15–30 min	Corn/Chicken, pork, beef/Wine	[14,15,40]
Colloidal Au-based ICA	10/6	15 min	Maize, wheat, rice/Corn, wheat, feedstuffs	[17,21]
Fluorescent -ICAs	0.1/1	20 min/8 min	Maize, wheat, vegetable oil/Maize	[19]/[20]
Aptamer-based lateral flow test strip	20	5 min	Corn	[41]
AuSP-ICA and AuNRs-ICA	5.0 and 3.0	10 min	Corn; wheat	This work

## Supplementary Materials

The following are available online at https://www.mdpi.com/2304-8158/9/3/281/s1. Table S1: The chemical structure of the tested mycotoxins in the research.
