# Supplementary material for: Development of Lateral Flow Immunochromatographic Assays Using Colloidal Au Sphere and Nanorods as Signal Marker for the Determination of Zearalenone in Cereals
Source: Foods. 2020 Mar 4;9(3):281. doi: 10.3390/foods9030281 (PMC7143912; doi:10.3390/foods9030281)

Table S1. The chemical structure of the tested mycotoxins in the research

Compounds	Chemical structure
ZEN	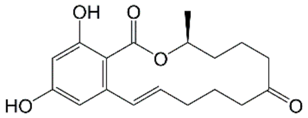
α-Zearalenol	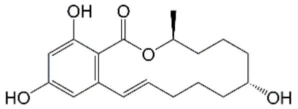
β-Zearalenol	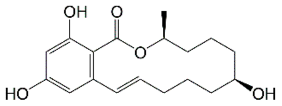
α-Zearalanol	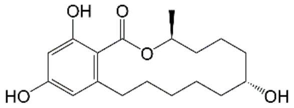
β-Zearalanol	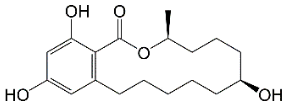
Zearalanone	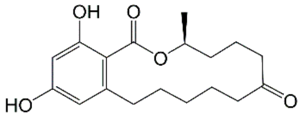
Aflatoxin B_1	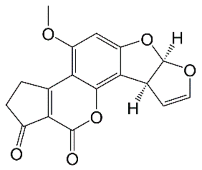
T-2 toxin	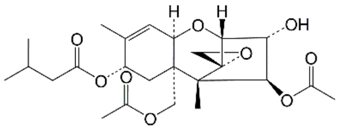
Ochratoxin A	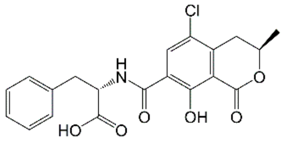
Deoxynivalenol	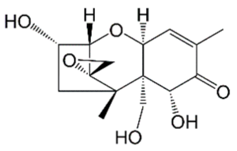

Fumonisin B_1

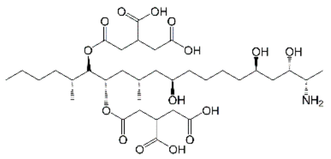

Supplement: Supplementary file 1 [file foods-09-00281-s001.pdf]
